# An fMRI study of visuo-vestibular interactions following vestibular neuritis

**DOI:** 10.1016/j.nicl.2018.10.007

**Published:** 2018-10-09

**Authors:** R.E. Roberts, H. Ahmad, M. Patel, Danai Dima, R. Ibitoye, M. Sharif, R. Leech, Q. Arshad, A.M. Bronstein

**Affiliations:** a Neuro-otology Unit, Division of Brain Sciences, Charing Cross Hospital, Imperial College London, London W6 8RP, UK; b Department of Neuroimaging, Institute of Psychiatry, Psychology and Neuroscience, King's College London, De Crespigny Park, Camberwell, London SE5 8AF, UK; c Department of Psychology, School of Arts and Social Sciences, City, University of London, London, UK; d De Crespigny Park, Camberwell, London SE5 8AF, UK; e Computational, Cognitive and Clinical Neuroimaging Laboratory, Division of Brain Sciences, Imperial College London, Hammersmith Hospital, London, UK

**Keywords:** fMRI, Visual-cortex, Oscillopsia, Vertigo, Vestibular compensation, Vestibular neuritis

## Abstract

Vestibular neuritis (VN) is characterised by acute vertigo due to a sudden loss of unilateral vestibular function. A considerable proportion of VN patients proceed to develop chronic symptoms of dizziness, including visually induced dizziness, specifically during head turns. Here we investigated whether the development of such poor clinical outcomes following VN, is associated with abnormal visuo-vestibular cortical processing. Accordingly, we applied functional magnetic resonance imaging to assess brain responses of chronic VN patients and compared these to controls during both congruent (co-directional) and incongruent (opposite directions) visuo-vestibular stimulation (i.e. emulating situations that provoke symptoms in patients). We observed a focal significant difference in BOLD signal in the primary visual cortex V1 between patients and controls in the congruent condition (small volume corrected level of *p* < .05 FWE). Importantly, this reduced BOLD signal in V1 was negatively correlated with functional status measured with validated clinical questionnaires. Our findings suggest that central compensation and in turn clinical outcomes in VN are partly mediated by adaptive mechanisms associated with the early visual cortex.

## Highlights

• VN clinical status related to V1 response to congruent visuo-vestibular stimuli
• Reduced V1 BOLD signal during congruent stimulation correlates with subjective dizziness scores
• No association between V1 BOLD signal and incongruent visuo-vestibular stimulation
• Changes in V1 activity may reflect cortical adaptive mechanisms following VN

## 1 Introduction

Acute vestibular neuritis (VN) is characterised by vertigo, nausea, postural instability and vestibular nystagmus (Dix and Hallpike, 1952; Strupp and Brandt, 2009). Recovery following VN is dependent upon both, regaining peripheral vestibular nerve activity and central compensatory processes, which together allow for the acute vestibular-ocular and vestibular-spinal signs to gradually dissipate over a few weeks (Curthoys and Halmagyi, 1995; Strupp and Brandt, 2009). Unfortunately, 30–50% of patients develop chronic symptoms of variable severity, including head movement and visually-induced dizziness (Cousins et al., 2013; Cousins et al., 2014; Cousins et al., 2017; Imate and Sekitani, 1993; Perols, 1999). Whether the development of such chronic symptoms following VN is predominantly mediated by peripheral or central mechanisms remains unclear. Recent data suggests that central mechanisms are more important as supported by, (i) a lack of association between the degree of functional inner-ear loss and symptom load (Best et al., 2006; Cousins et al., 2017; Palla et al., 2008; Patel et al., 2016) and, (ii) the degree of visual reliance during central integration of sensory cues (“visual dependence”) (Cousins et al., 2017), and selective vestibulo-perceptual deficits (Panichi et al., 2017) closely predicting clinical outcome following VN.

Visuo-vestibular symptoms associated with head turns in VN patients (e.g. dizziness, oscillopsia, spatial disorientation) partly relate to disruption of gaze stability. Normally, the vestibular-ocular reflex (VOR) generates most of the slow phase eye movement required for image stabilization during head turns, with only minor contributions by the optokinetic system. After acute vestibular loss, the normal vestibular-optokinetic apportionment is drastically reversed (Farhat et al., 1995), and consequently patients are highly symptomatic during head turns (Strupp and Brandt, 2009). Accordingly, the ability to deal with this head movement-induced visuo-vestibular mismatch may partly determine which VN patients proceed to develop chronic symptoms.

Studies in healthy subjects have investigated the interaction between visual and vestibular stimuli using neuro-imaging (Billington and Smith, 2015; Frank et al., 2016; Roberts et al., 2017). Specifically, our recent fMRI study (Roberts et al., 2017) combined caloric stimulation and visual motion to study the cortical interactions between vestibular and visual signals. We demonstrated that when vestibular (caloric) and visual (optokinetic) stimuli are congruent (i.e. the slow phase eye movement induced by both stimuli is co-directional; as during conventional head turns), there was increased activation in the primary and secondary visual cortices. During incongruent stimulation (i.e. the eye response is in opposite directions; an unfamiliar infrequent situation), there was a preferential activation of multisensory vestibular cortical areas including the posterior insular cortex, which may play a role in disambiguating visual and vestibular cues. Based on these results in healthy controls, and that during daily life VN patients must deal with mismatched visuo-vestibular signals during head movements, we postulate that the brain responses of chronic VN patients will differ to controls.

In support of our proposition are previous neuroimaging studies in VN (reviewed by Dieterich and Brandt) (Dieterich and Brandt, 2010) which have revealed changes in visual cortical areas and vestibular cortical networks (Helmchen et al., 2009; Helmchen et al., 2014). Furthermore, increases in grey matter density after VN have been reported in visual cortical areas (Zu Eulenburg et al., 2010), superior temporal gyrus (STG), insular, cerebellum and inferior parietal lobe (Helmchen et al., 2009) (Hong et al., 2014). Notably, the changes in the STG and cerebellum are associated with more marked improvement following vestibular loss. Moreover, studies that have compared brain activity differences between the acute and chronic stages of VN, have found that vestibular areas were more active compared to visual areas (Bense, Bartenstein, et al., 2004), with the laterality of these effects dependent upon the side of the lesion (Becker-Bense et al., 2014). Finally, functional resting state analysis suggests that functional outcome might rely on the restitution of connectivity associated with the intraparietal sulcus (Helmchen et al., 2014). Taking together the evidence from both structural and functional imaging studies implies that brain changes following VN occurs rapidly, and in the same regions as the reported activations during vestibular stimulation in healthy individuals (Suzuki et al., 2001; Fasold et al., 2002; Naito et al., 2003).

However, whether the brain response of VN patients differs from controls during combined visuo-vestibular stimulation remains unknown. Accordingly, we now explore this using fMRI by concurrently stimulating the visual and vestibular systems in both the same direction (i.e. mimic normal head turn - congruency frequently experienced in daily life) and opposite directions (i.e. visuo-vestibular incongruence). As aforementioned, our study in young healthy individuals identified two primary areas of activation associated with either congruent or incongruent combined visuo-vestibular stimulation. That is, congruent visuo-vestibular stimulation predominantly activated early visual cortical areas, whereas incongruent activation was associated with the posterior insular region (Roberts et al., 2017) and, based on these findings, we restricted our analysis to test for differences confined to these regions. We predict greater differences in patients compared to healthy controls in visual areas during congruent stimulation, and vestibular areas during incongruent stimulation. Furthermore, we postulate that if a patient's clinical outcome is related to their degree of adaptation to lesion-induced mismatched visuo-vestibular signals, then one would also expect individual differences in BOLD signal response to correlate with symptom severity.

## 2 Materials & methods

Written informed consent was obtained from all participants. All procedures performed were in accordance with the ethical standards of the Bromley and the Fulham local research ethics committee.

### 2.1 Participants

In order to control for laterality, we recruited 17 chronic (>6 months' post-onset) (N.B. sample size derived from previous studies investigating cortical changes following VN (Becker-Bense et al., 2014) right-sided VN patients (mean age 58.8, SD = 17.3, 8 males). All participants were right-handed, thus avoiding confounds associated with handedness-related vestibular hemispheric dominance (Arshad et al., 2015; Arshad, 2014; Arshad et al., 2013a; Dieterich et al., 2003; Nigmatullina et al., 2016). All patients in the acute stage had acute vertigo, spontaneous horizontal-torsional nystagmus, a positive head impulse test and no other neurological/audiological symptoms (Baloh, 2003). Formal testing revealed a canal paresis (30-44 °C caloric testing; mean CP% = 68, range 20–100%) (Cousins et al., 2017; Karlberg and Magnusson, 2011) and normal-for-age hearing. Seventeen right-handed matched controls were recruited (mean age = 53.4, SD = 19.5) with no history of labyrinthine (confirmed with caloric testing in the laboratory) or neurological disorders.

## 3 Clinical questionnaires

Participants completed three questionnaires to assess symptom load (Table 1). This included the (i) vertigo symptom scale (VSS – assessing the frequency and severity of dizziness symptoms) (Yardley et al., 1992), (ii) situational vertigo questionnaire (SVQ - providing a score of the severity of symptoms induced by visually disorienting environments) (Jacob et al., 1989; Jacob et al., 1993), and (iii) dizziness Handicap Inventory (DHI – assessing both physical and emotional symptoms as well as the degree of dizziness related functional impairment) (Jacobson and Newman, 1990).

**Table 1:** Demographic details of patient and control groups. Percentage canal paresis in acute stage (CP%), vertigo symptom scale (VSS), dizziness handicap inventory (DHI), situational vertigo questionnaire (SVQ). *Indicates *p* < .01 between group differences.

**PATIENTS**						
**ID**	**Age**	**Sex**	**CP(%)**	**VSS**	**DHI**	**SVQ**
1	55.5	M	66	25	70	28
2	71.4	F	100	17	24	8
3	67.6	M	82	0	0	0
4	80.5	M	49	0	24	14
5	67.7	M	100	0	0	0
6	27.1	F	27	34	60	0
7	55.3	M	20	0	0	0
8	70.2	M	28	20	64	5
9	45.2	F	37	0	12	0
10	44.4	M	62	14	20	24
11	61.0	F	39	14	50	23
12	75.8	F	61	0	12	18
13	47.4	F	28	42	54	33
14	58.3	M	100	14	33	12
15	20.7	F	28	1	32	0
16	74.9	F	100	1	10	1
17	76.2	F	25	52	94	45
**Mean**	**58.8**	**8** **M**	**56.0**	13.8*	32.9*	12.4*
**SD**	**17.3**	**–**	**30.3**	**16.4**	**27.9**	**14.0**

**CONTROLS**						
**Mean**	**57.5**	**7** **M**	**–**	**2.5**	**0.59**	**1.5**
**SD**	**14.1**	**–**	**–**	**4.5**	**2.4**	**3.1**

Bold indicates P< 0.01

### 3.1 Psychophysical measures: visual dependency

**Fig. 1:**
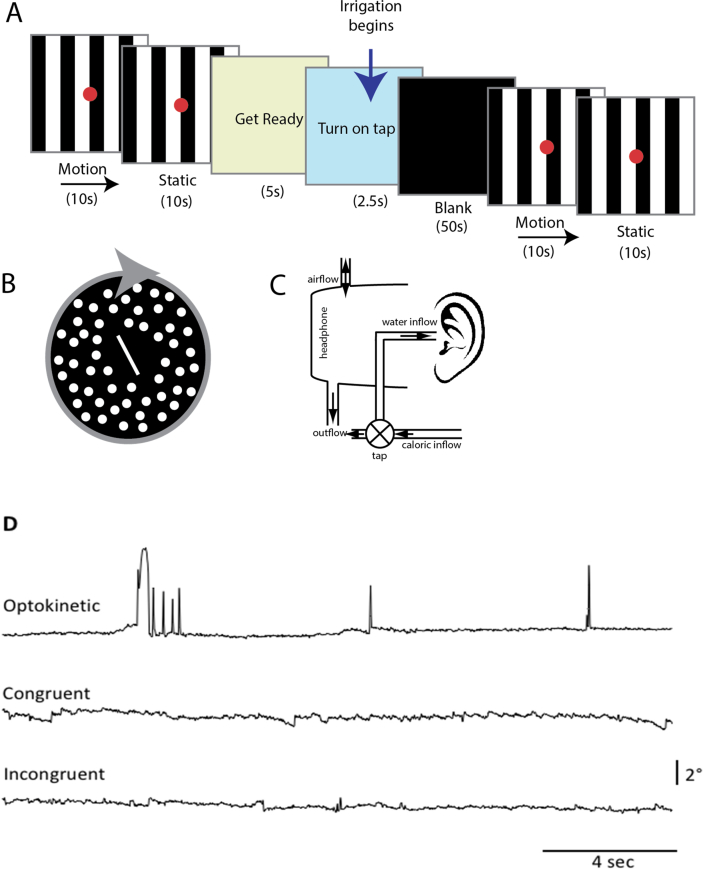
A Experimental Design and Apparatus Schematic of stimulus used in visuo-vestibular interaction experiment. (B) Psychophysical stimulus used to measure subjective visual vertical (while background is static) and visual dependency (background rotating in roll plane). (C) Experimental apparatus for irrigating the left-ear canal inside the MRI scanner. Circulating water was diverted into the left-ear canal via a manually operated tap, controlled by the participant. The water exits via the outflow pipe and the pressure is equalised by the airflow inlet. (D) Examples of eye movement recordings of a subject in the scanner. During the ‘Optokinetic’ sequence shown the stripes drifted to the left. ‘Congruent’ stimulation was achieved by combining left visual motion and left ear cold water irrigation. During the “Incongruent” stimulation shown visual motion to the right was combined with left ear cold water irrigation. Only during “Congruent” stimulation a small nystagmus could be recorded (right beating nystagmus in the example shown).

Given that poor clinical outcome in vestibular disorders is associated with high levels of visual dependence (how much weight an individual gives to visual input for spatial orientation), we measured visual dependence with the Rod-and-Disk Test **(Fig.1B)**.(Cousins et al., 2014, Cousins et al., 2017) This involved participants seated upright watching a laptop screen through a viewing cone that excluded extraneous visual orientation cues, subtending a visual angle of 39 degrees. The visual stimulus consisted of a white 6 cm rod on a black background. Outside of this central zone, the viewing screen was filled with off-white dots, each subtending 1.5 degree of visual field, randomly distributed on a black background. Subjects were instructed to align the rod to their perceived vertical in three conditions: stationary dots; dots rotating clockwise (30 degrees/s) or anticlockwise (randomised order; 4 trials performed per condition). The rod was initially set to ±40 degrees from vertical. For each trial the difference in degrees between true vertical and the subjects' final rod placement was calculated. Visual dependence was calculated as the mean tilt deviation during motion minus the static measure. Software available at: http://www.imperial.ac.uk/medicine/dizzinessandvertigo.

## 4 Stimuli during fMRI

### 4.1 Visual stimuli

The visual stimuli consisted of eight alternating black or white stripes, each subtending an angle of 1.9 degree, on a screen with a visual angle of 15 degree. The stimuli conditions were either stationary, or moving optokinetic stimulus (OKS), either left or rightwards with a velocity of 8 degree/s, superimposed with a central red fixation dot subtending 0.5 degrees. At the beginning of each run there was a 60s baseline period of visual stimuli with three 10s periods of static and three periods of visual motion **(**Fig. 1**)**, in a counterbalanced order. Each condition was presented for a period of 10s, six times in total **(Fig. 1B)**.

### 4.2 Vestibular stimuli

To provide vestibular stimulation within the scanner, a modified headset with inlet and outlet tubes was developed, **(Fig. 1C**; also see Roberts et al., 2017 for details**)**. A nozzle was positioned in the left-ear canal secured with surgical tape all housed in headphones, connected via a thermally insulated tube (with a continuous circulation system to maintain temperature) to the irrigation system (ICS medical). The head was positioned 30 degrees above the horizontal to ensure maximal vestibular activation, and to minimize the influence of magnetic fields stimulating the vestibular system (Roberts et al., 2011). Irrigations were performed in dim illumination with no visual stimulus, for 50s with 250 ml of either ‘cold’ (30 °C) or ‘warm’ (44 °C) water. Each participant received two cold and two warm irrigations in a counterbalanced order, always of the left-ear (recall all VN patients were right-sided). Participants were cued to self-initiate irrigations by turning a hand-held tap to minimize participant discomfort (N.B. a delay of four volumes was employed between tap on and data acquisition, to ensure no residual motor activation), and any potential head movements which can occur when caloric irrigation begins (N.B. no participant reported sickness following caloric irrigations). Immediately following the irrigations, the visual stimulus was presented for 60s. Throughout, participants kept their eyes open in order to record eye movements using an infrared MRI-compatible eye tracking system **(Fig. 1D)** (Ober consulting, Poland).

### 4.3 fMRI experimental Design and Analysis

Four experimental runs were performed with each run lasting approximately 3.5 min. A block design was implemented with two factors; TEMPERATURE of left ear caloric irrigation (i.e. cold or warm), and DIRECTION of visual motion (i.e. left or rightwards). These conditions were grouped as in Roberts et al. (2017) to provide conditions where the slow-phase eye movements elicited by each of these stimuli in isolation would be in the same direction (“congruent”, i.e. right cold irrigation + rightward motion or right warm irrigation + leftwards motion) or where the slow-phase eye movements would be in opposite directions (“incongruent” condition i.e. right cold irrigation + leftwards motion or right warm irrigation + rightward motion). Implementing such a design allowed us to control for any potential differences attributable to stimulation temperature, somatosensory stimulation and nystagmus direction, which have all previously been shown to induce differential brain activation (Bense et al., 2006; Dieterich et al., 2003; Naito et al., 2003). The peak vestibular response derived from the peak slow phase velocity of the eye movements during irrigation was used as a covariate in the subsequent MRI analysis to account for inter-individual differences in vestibular activation. At the end of each run participants were asked to rate their subjective experience of dizziness on a Likert scale, rating the intensity of the standard caloric they received as part of the screening process as a ‘5’ on the scale. This measure was included as a nuisance covariate in the final analysis stage.

### 4.4 First level analysis

For image pre-processing and statistical analysis, we used the SPM8 software package (Wellcome Trust Centre for Neuroimaging, London, UK: www.fil.ion.ucl.ac.uk). For each participant the data from the four conditions was concatenated and modelled with a general linear (convolution) model with movement parameters included as confounds. Vectors representing the onset of visual motion, visual static and caloric onsets were convolved with a hemodynamic response function. Based on our recent findings in young healthy individuals with the same experimental protocol, we employed an ROI approach (using the V1 mask from our previous study) and employed a small volume correction threshold where *p*-values *p* < .05 (FWE) were considered statistically significant to test for differences in activation within insular (4256 voxels) and primary visual cortex (732 voxels; BA17) (Roberts et al., 2017). In a secondary, exploratory analysis we also tested for differences in V5/MT (3775, BA19) and parahippocampal place area (PPA, 4072 voxels). These regions were masked using SPM8's Marsbar toolbox (http://marsbar.sourceforge.net). All results are reported in MNI coordinates.

Additional TRs were then taken to construct the 30s periods of static or moving visual periods. A high-pass filter (128 s) was employed to remove low frequency noise, and serial correlations were removed using a first-order auto-regressive model. An explicit mask was used to include only voxels within the brain as part of the analysis. The analysis focused primarily on the interaction between visual motion stimuli immediately following the caloric irrigation when participants were experiencing caloric-induced vertigo. Thus, we compared activation during baseline (no vertigo) with the post caloric period.

### 4.5 Second level analysis

Group-level analyses were based on random-effects analyses of the single-subject contrast images using the summary statistic approach. Independent sample *t*-tests were used to investigate group differences during both congruent and incongruent conditions separately.

### 4.6 Image acquisition and processing

Gradient echo planar MR images were acquired on a Siemens Verio 3 T scanner. For each participant, four runs were performed with caloric irrigation (99 volumes) and one run used as a visual localiser (96 volumes). Functional T2*-weighted images were acquired at each of 44 axial, contiguous planes using a gradient echo sequence in an interleaved order (TR = 2500 ms, TE = 30 ms, flip angle = 80^o^, voxel dimensions = 3 × 3 × 3 mm, acquisition matrix = 64 × 64). For each participant a high-resolution T1-weighted anatomical image was acquired in the axial plane for subsequent co-registration (TR = 2300 ms, TE = 3 ms, TI = 900 ms, Flip angle = 9^o^, Bandwidth = 238 Hz/pixel, voxel dimensions 1 × 1 × 1 mm, matrix size = 256 × 192, FOV = 240 × 256 mm, slice thickness = 1 mm, Number of excitations = 1). The T1 images were inspected for structural abnormalities and scored for deep white matter hypointensity using the Fazekas scale by a neurologist. Two patients but no controls had single lacunar lesions. No other abnormalities were seen. Volumetric images were processed using FSL brain extraction and segmentation tools with results inspected for accuracy. Separately, a neuro-radiologist reported the scans for significant incidental findings but none were identified. Foam padding was used to limit head motion. For image pre-processing and statistical analysis, we used the SPM8 software package (Wellcome Trust Centre for Neuroimaging, London, UK: www.fil.ion.ucl.ac.uk). We used an ROI approach and employed a small volume correction threshold where *p*-values *p* < .05 (FWE) were considered statistically significant. Images were realigned to correct for movement and normalised into MNI space using each subject's structural MRI image. The data were then smoothed with an 8 mm Gaussian filter (FWHM).

## 5 Results

### 5.1 Imaging findings

**Fig. 2:**
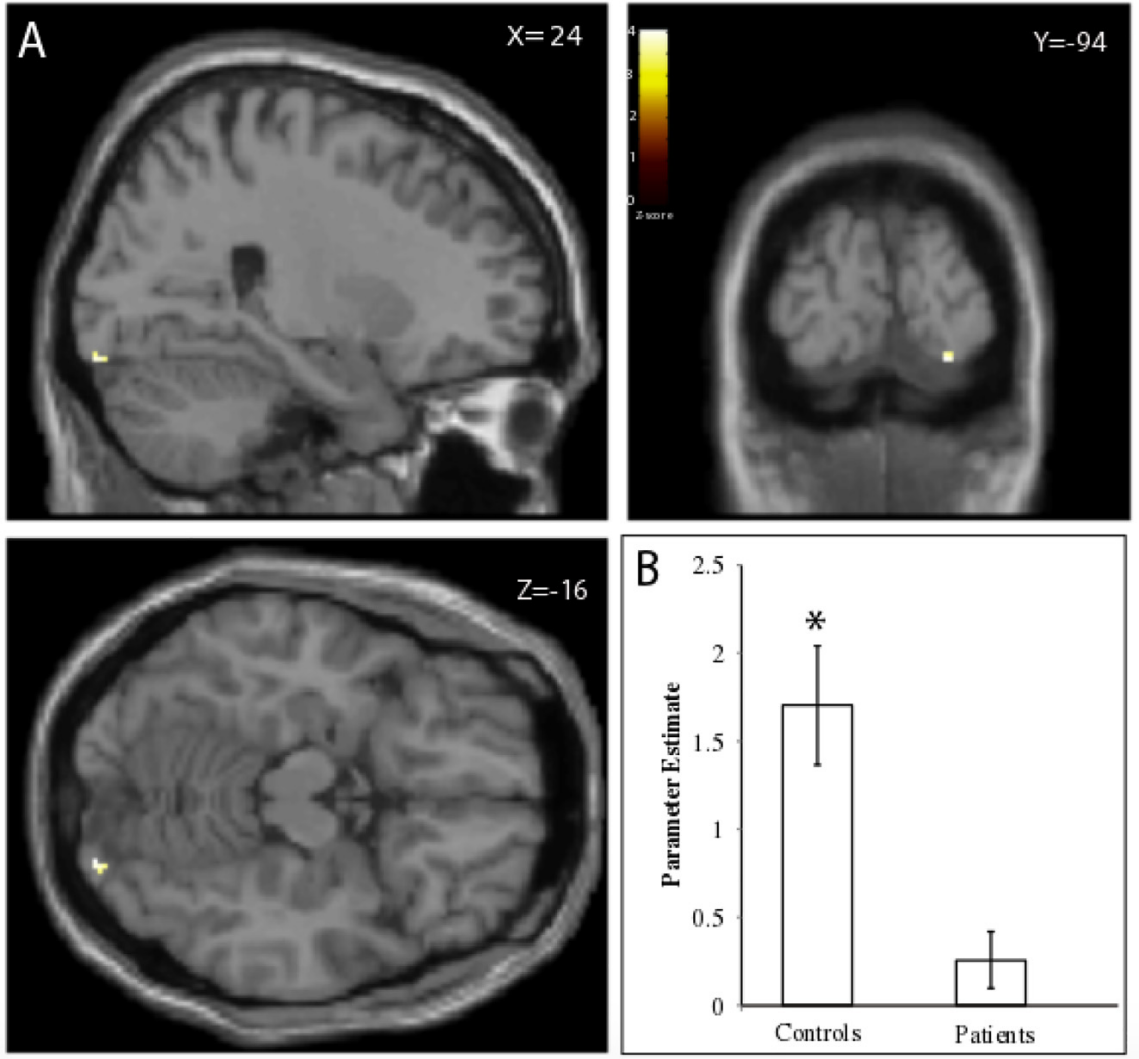
Differences in brain activation between patient and control groups. (A) We observed a significant difference in brain activation within visual cortical areas V1 between the patient and control groups in the congruent condition. (B) The patients exhibited reduced activation in the congruent condition. All activations are superimposed on a canonical single subject T1 structural image template. All coordinates are in MNI space. Heat bar indicates *Z*-statistic.

Based on our recent findings in normal subjects (Roberts et al., 2017) we compared VN patients vs. control subjects in the defined ROIs (see Introduction and Methods). We grouped the visuo-vestibular conditions into two, either congruent or incongruent. During the congruent condition we observed a significant difference between the patient and control groups within a focal area (20 voxels) of the right V1 visual cortex (*T* = 4.31; *Z*-value = 3.77; X = 24, Y = −94, Z = −16; *p* < .04, FWE) **(**Fig. 2A**)**. That is, patients exhibited significantly reduced activation compared to the controls **(**Fig. 2B**)**. However, no group differences were observed during the congruent condition within the posterior insular ROI. Furthermore, there was no significant difference between the groups during the incongruent condition in any of the ROIs.

In a secondary set of exploratory analyses, we tested for group differences in V5/MT and PPA. We did not observe any significant activation, although lowering the threshold (to *p* = .01 uncorrected) revealed V5/MT activation. A further whole brain analysis for each contrast was also conducted as part of a secondary results stage. This revealed no significant activations (whole brain corrected, *p* > .05) (See supplemental data). Although our principal approach was to use ROIs based on our previous findings, these secondary analyses demonstrate that there was relatively little involvement of other cortical networks using these contrasts.

### 5.2 Relationship between imaging findings and clinical questionnaires

**Fig. 3:**
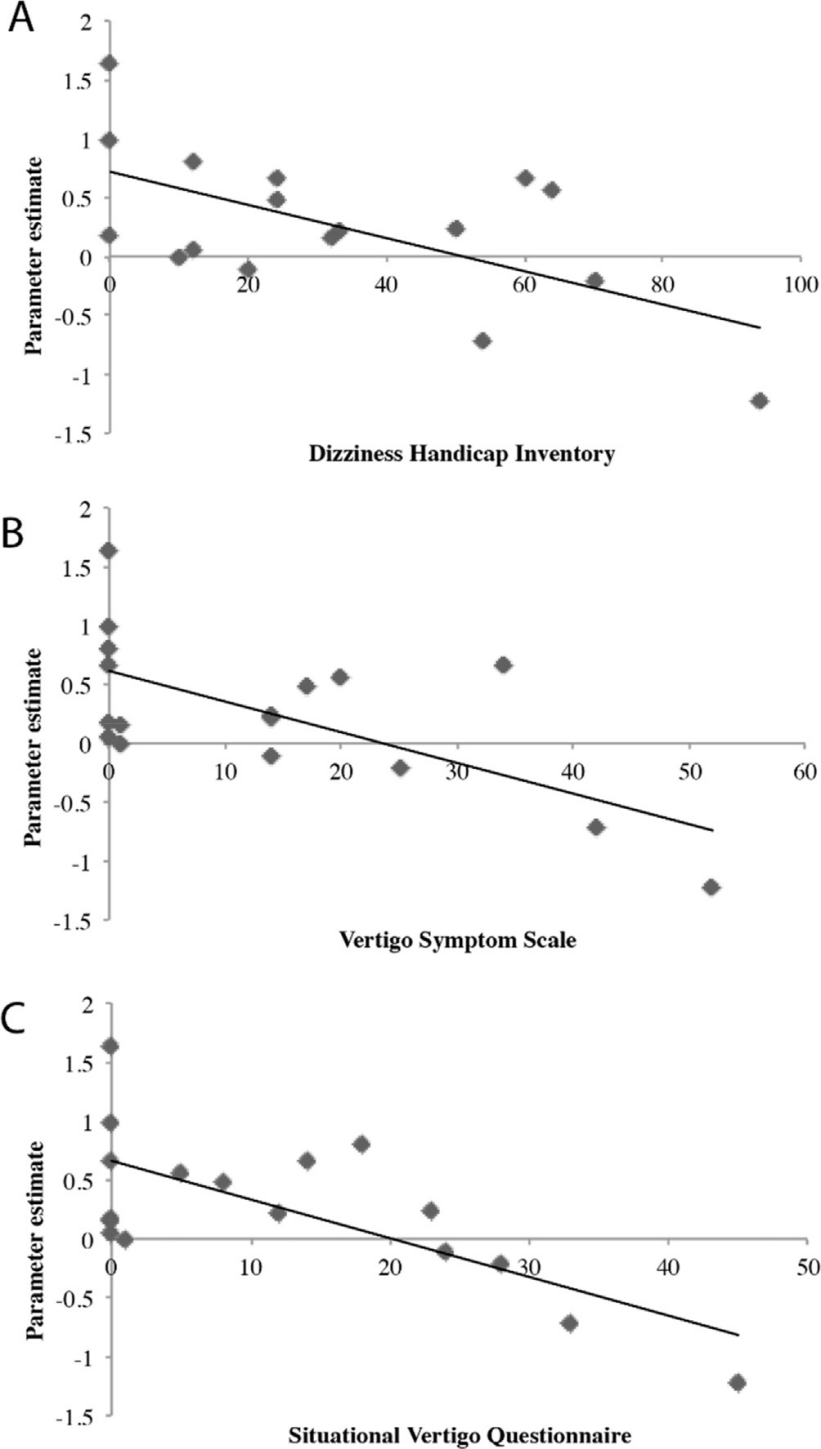
Associations between clinical status and brain activity within visual cortex (A-C). We observed significant negative associations between clinical functional status in the patient group, as indexed by questionnaires, and the time course of activation within the peak voxel in the congruent condition across the patient group (parameter estimate, arbitrary units).

Based on these results we extracted the time course of the peak voxel of activation in the focal area of the right V1 in the patient group during the congruent condition and assessed the relationship with self-reported symptom load as assessed by the questionnaires. This revealed significant correlations with all clinical questionnaires, VSS (*r* = −0.65, *p* = .004), SVQ (*r* = −0.71, *p* = .002) and DHI (*r* = −0.60, *p* = .010) **(**Fig. 3A-C**)**. We also assessed the relationship with two principal subscales in the VSS for vertigo symptoms (mean = 5.8, SD = 7.4) and autonomic/anxiety (mean = 8.0, SD = 10.1), as well as the functional (mean = 13.2,SD = 11.3), physical (mean = 10.5,SD = 8.1) and emotional (mean = 9.2,SD = 10.1) subscales of the DHI. There was a significant correlation with vestibular symptoms (r = −0.65, *p* = .005) and borderline significance with anxiety/autonomic symptom (−0.56, *p* = .02) subscales of the VSS, and a significant correlation with the functional (*r* = −0.67, *p* = .003) but not physical (*r* = −0.5, *p* = .16) or emotional handicap (*r* = −0.51, *p* = .14) subscales of the DHI. We used a bonferroni corrected *p*-value (=0.01) to adjust for the number of primary and secondary clinical outcome associations we tested.

### 5.3 Relationship between imaging data and psychophysical measures

**Fig. 4:**
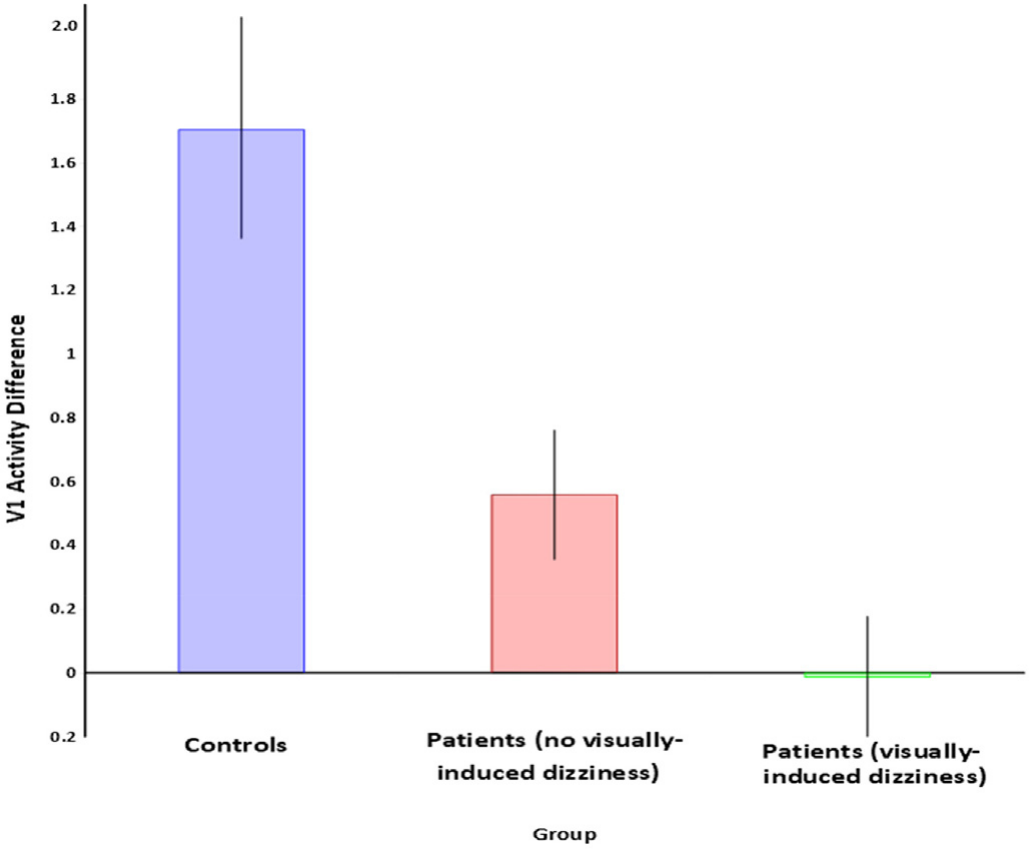
Control and patient group activity in V1. Mean group activity for V1 is presented for (a) controls, (b) patients with vestibulopathy and no visually-induced dizziness, and (c) patients with both vestibulopathy and visually-induced dizziness. Both patient groups were significantly different to controls, and there was a non-significant trend towards a difference between the two patient groups.

Brain activity and visual dependency (visual motion induced tilt of the subjective visual vertical rod, in degrees) were not significantly associated (*r* = 0.1, *p* = .73). This may reflect the fact that in this particular patient group, the association between visual dependency and clinical status did not quite reach statistical significance: VSS: *r* = −0.4, p = .1; DHI: *r* = −0.33, *p* = .19. To further probe this surprising lack of relationship, we divided the patient group into those who reported virtually no symptoms on the VSS (a score of 1 or 0), and those with higher scores and significant visually-induced dizziness (visual vertigo) **(**Fig. 4**)**. The average activation in V1 for non-visually induced dizzy patients was 0.56, SD 0.57 and for visual vertigo patients was −0.013, SD 0.63 (arbitrary activation units). These were both found to be significantly different to the controls, (*p* < .05). We also tested for differences in the means between the two patient groups, (independent samples *t*-test), which was at the trend level of significance (*p* = .07). This may suggest that with larger samples these groups would be statistically separable based on V1 activity.

### 5.4 Physiological variables and their relationship with imaging data

Age was not associated with the response in the visual cortex (*r* = 0.04, *p* = .9). Formal assessment of the degree of vestibular activation assessed using (i) slow phase eye movement velocity revealed no differences between conditions F(2.8, 90.1) = 0.14, *p* = .93 or subject group F(1,32) = 0.017, p = .9 and, (ii) caloric-induced dizziness intensity with a Likert scale (Roberts et al., 2017) showed no differences between conditions (F(2.7, 87.4) = 0.82, *p* = .47) and subject group (F(1,32) = 1.65, *p* = .21) (N.B. recall that the patients had their healthy ear irrigated). Patients' age and degree of canal paresis were not correlated with any of the clinical questionnaire (age; *r* = 0.02, *p* > .05; canal paresis; r = 0.04, p > .05) or imaging data (age; *r* = 0.03, p > .05; canal paresis; r = 0.03, p > .05). These findings are in agreement with our recent papers showing that age, degree of canal paresis or abnormalities in the video head-impulse test were not correlated with clinical outcome (Cousins et al., 2017; Palla et al., 2008; Patel et al., 2016).

### 5.5 Relationship between eye movements and imaging data

An outstanding possibility is that the reported effects observed in the visual cortex may be attributable to eye movement related effects, despite the large suppression afforded by the central fixation dot. Accordingly, we measured both, (i) mean variance in eye position and (ii) peak slow phase eye velocity in the horizontal plane for each participant and tested for any correlation with V1 activity This analysis did not indicate any significant relationship (p > .05) for either mean variance in eye position (r = 0.02) or peak slow phase eye velocity respectively (*r* = 0.012).

### 5.6 Influence of small vessel disease

Finally, in view of recent reports that the amount of small vessel disease can have an impact on outcome of VN (Adamec et al., 2014) or induce dizziness in the elderly (Ahmad et al., 2015) we decided to examine this in our data. Seven patients were scored non-zero on the Fazekas scale. Fazekas scores and mean brain grey, white and total brain volumes did not differ significantly between patient and control groups (Chi squared test; all *p* values >.05). We then tested for associations between clinical status and brain morphology in the patient group. We split the group (7 vs 10) into patients with a Fazekas of 0, and those with Fazekas >0. Although we found no significant differences (independent samples *t*-test), DHI (*p* = .06) and VSS (*p* = .07) showed a trend towards significance. We also tested for correlations between total brain volume and clinical scales, which were non-significant (*p* = .085). This may well reflect a lack of power due to the group size being split (7 vs 10).

## 6 Discussion

We observed that congruently combined visuo-vestibular activation differentially modulated cortical responsiveness in patients with VN compared to controls. Specifically, we observed a significant difference between patients and controls in the condition when the visual and vestibular stimuli signalled self-motion in the same direction (i.e. congruently). Patients displayed reduced activation of the primary visual cortex (V1), suggesting that this area plays a role as an adaptive mechanism in supressing visuo-vestibular symptoms. We proceeded to examine how the variability of activation within this focal region (N.B. observed at a small volume FWE-corrected level of *p* < .05) was related to clinical functional status by correlating brain activation with validated questionnaires assessing symptoms and functional impairment. We observed that those patients with the lowest level of BOLD signal change in V1 reported the most profound symptoms.

Previous neuroimaging studies in VN patients have predominantly focused on structural changes and reported effects in both visual (Zu Eulenburg et al., 2010), and vestibular cortical areas (Helmchen et al., 2009). Changes in the vestibular cortical network have been associated with the functional status (Helmchen et al., 2009), however it is important to note that functional changes do not necessitate structural changes, particularly if a brain region is regulated by a secondary area. Henceforth, our finding in V1 might equally be due to modulation by higher-order visual areas (V5/MT) or attentional mechanisms associated with the posterior parietal cortex (Antal et al., 2003; Arshad et al., 2013b; Silvanto et al., 2008).

Extending on the aforementioned research, our study in VN patients adds knowledge by demonstrating the brain's response to dynamic interactions between visual and vestibular stimuli. This is particularly relevant because chronic VN patients do not experience symptoms whilst lying down or inactive, however they do report problems during conditions of active head movements as experienced in daily life. This was simulated herewith by our experimental paradigm which revealed that congruent combined visuo-vestibular stimulation that simulates head turns in a normal environment modulates BOLD signal in the early visual cortex.

Although perhaps speculative, the differences observed in primary visual cortex can be interpreted in the light of recent neuro-physiological findings in bilateral vestibular patients (Ahmad et al., 2017). In that study, using transcranial magnetic stimulation of the visual cortex, down-regulated excitability of the early-visual cortex was associated with reduced oscillopsia-related handicap in bilateral vestibular patients (i.e. less clinical impact of the abnormal movement of visual images). The extent of the observed modulation in excitability was associated with functional outcome (Ahmad et al., 2017). Here we observed reduced V1 BOLD signal change in the more symptomatic VN patients which, when taken together with the neuro-physiological data in bilateral patients, lends support to the notion that the primary visual cortex plays an important role in suppression of visuo-vestibular symptoms (an important adaptive requirement for vestibular patients). Physiologically, the oscillopsia in bilateral vestibular patients is due to the slippage of the retinal image during head movements, particularly noticeable during locomotion. Despite VN patients not experiencing significant oscillospia during locomotion, there is a core similarity between the process of recovery in unilateral (VN) and bilateral vestibular patients. The low VOR gain present on turning the head towards the side of the lesion generates considerable retinal slip in VN patients and, reciprocally, retinal slip during head motion is undoubtedly the mechanism underlying locomotion oscillopsia in bilateral vestibular patients. Hence, it seems likely that the V1 fMRI findings in unilateral VN patients and the V1 TMS findings in bilateral vestibular failure reflect the process of adaptation to low VOR gain and retinal slip, as observed in both clinical conditions. In support, the trend illustrated in Fig. 4 shows that those VN patients with visually induced dizziness had greater differences in V1 BOLD signal compared to both controls and patients with no visually induced dizziness.

The reduction in BOLD signal change in V1 in our patients was confined to the condition where the visual and vestibular stimuli were co-directional (congruent). This visuo-vestibular experience simulates head turns in the real world, and this is reflected in our previous psycho-physical finding with an identical paradigm where 9/10 subjects chose this condition as the one most closely reflecting real-world motion (Roberts et al., 2017). This phenomenon occurred despite the suppressed nystagmic eye movements because, both in the present and the previous study, a screen fixation dot was used in order to diminish eye movement-related confounds (Roberts et al., 2017). Thus, combined congruent visuo-vestibular processing is the condition most frequently experienced in daily life and, critically, the condition to which VN patients must learn to adapt. It is possible that over time this adaptation process is responsible for inducing the observed difference between healthy controls and patients.

In the “conflict” or incongruent condition no differences were observed between the two groups. This may reflect the unusual combination of stimuli, in which the vestibular system signals motion in one direction and the visual system in the opposite. This is less commonly experienced and furthermore infrequently recognised by participants as a natural scenario (Roberts et al., 2017). In healthy controls, we have previously shown that such conflict elicits a pattern of fMRI activation of primarily vestibular and posterior insular cortices. This may reflect a system involved in disambiguating visuo-vestibular conflict by activation of vestibular (inertia-driven) cortical areas (Roberts et al., 2017). In this light, the lack of patient-control differences in the conflict (incongruent) condition could simply mean, (i) that the condition is unusual for both subject groups and/or, (ii) that suppressing visuo-vestibular conflict and motion sickness is actually easier when vestibular function declines (Murdin et al., 2015).

The results of the present study demonstrated no relationship between V1 bold activity and visual dependence. This was surprising given that increased visual dependence, measured with the rod-and-disc task, has been found to be associated with poorer clinical outcome in VN patients. Recent research shows that visual dependence predicts outcome in VN as a single component in a factor analysis that includes psychological and behavioural variables (anxiety, arousal, somatization trends) (Cousins et al., 2017). In agreement with this point, the psychological variables represented in the current study as sub-scales of the DHI/VSS questionnaires did co-vary with fMRI activity in V1. Why visual dependence per se is not associated with V1 activity is not entirely clear but it must be kept in mind that visual dependence (as measured with the rod-and-disc task) is an extreme form of visuo-vestibular conflict, whereas our current finding is that VN patients and controls differ not during incongruent but during congruent visuo-vestibular stimulation. By extension then, it seems that visual dependence (and perhaps other forms of visuo-vestibular conflict) do not localise to visual cortical areas, something that we also found in an independent neuro-physiological study (Lubeck et al., 2016). A possible limitation of our study is that the only measure of vestibular function we used for correlations with fMRI signal change was the caloric test and not the quantitative head impulse test. Indeed, it is the case that caloric stimulation assesses the low frequency response of the VOR, whereas the head impulse test measures the high frequency response of the VOR. However, this issue is unlikely to be critical because, although bold signal change in V1 is associated with clinical outcome, the latter has been shown not to be correlated to quantitative head impulse results (Patel et al., 2016, Palla et al., 2008).

A final note about our results is related to the laterality of any brain changes that occur following VN, which are inherently complex to interpret. This is attributable to the fact that they arise from a complex interplay between lesion side, stimulation side and the handedness of the individual (Becker-Bense et al., 2014). To control for this, in our study we included only right-handed individuals with right sided VN, which is likely to explain the lateralised activation to the right visual cortex (Dieterich et al., 2003; Nigmatullina et al., 2016).

In conclusion, top-down modulation of the primary visual cortex may be a key component of effective adaptation following unilateral and bilateral peripheral vestibular loss. This is in line with clinical trial data indicating better clinical outcomes when visual stimulation is added to rehabilitation programs aimed at promoting patient adaptation (Pavlou et al., 2012; Pavlou et al., 2004; Whitney et al., 2016). Future studies may wish to consider how direct and indirect interventions to modulate activity in this brain area could be implemented to improve patient symptoms.

## Appendix A Supplementary data

Supplementary material Image 1
