# Supplementary material for: An fMRI study of visuo-vestibular interactions following vestibular neuritis
Source: Neuroimage Clin. 2018 Oct 9;20:1010–7. doi: 10.1016/j.nicl.2018.10.007 (PMC6197146; doi:10.1016/j.nicl.2018.10.007)
Supplement: Supplementary file 1 — Supplementary material [file mmc1.docx]

**Supplementary Table 1**

| **Controls Visual Main Effects** | | |  |  |  |  |  |
| --- | --- | --- | --- | --- | --- | --- | --- |
|  |  |  |  |  |  |  |  |
| **cluster** | **peak** | **peak** | **peak** | **peak** | **peak** |  |  |
| **equivk** | **p(FWE-corr)** | **p(FDR-corr)** | **F** | **equivZ** | **p(unc)** | **x,y,z {mm}** | **Region** |
|  |  |  |  |  |  |  |  |
| 363 | 0.001 | 0.111 | 58.29 | 5.60 | 0 | -32 -26 56 | Left Cerebrum, Frontal Lobe, Precentral Gyrus, Gray Matter, Brodmann area 4 |
|  | 0.002 | 0.111 | 51.95 | 5.39 | 0 | -40 -18 54 | Left Cerebrum, Frontal Lobe, Precentral Gyrus,  Gray Matter, Brodmann area 4 |
|  | 0.003 | 0.116 | 50.74 | 5.35 | 0 | -38 -42 60 | Left Cerebrum, Parietal Lobe, Postcentral Gyrus,  Gray Matter, Brodmann area 5 |
| 52 | 0.001 | 0.111 | 55.24 | 5.50 | 0 | 20 -60 64 | Right Cerebrum, Parietal Lobe, Superior Parietal Lobule, Gray Matter, Brodmann area 7 |
|  | 0.017 | 0.499 | 40.07 | 4.93 | 0 | 16 -56 70 | Right Cerebrum, Parietal Lobe, Postcentral Gyrus,  Gray Matter, Brodmann area 7 |
| 25 | 0.002 | 0.111 | 52.44 | 5.41 | 0 | -14 -66 62 | Left Cerebrum, Parietal Lobe, Superior Parietal Lobule, Gray Matter, Brodmann area 7 |
| 70 | 0.002 | 0.111 | 51.89 | 5.39 | 0 | -50 -32 52 | Left Cerebrum, Parietal Lobe, Postcentral Gyrus,  Gray Matter, Brodmann area 40 |
|  | 0.009 | 0.279 | 43.70 | 5.08 | 0 | -46 -40 48 | Left Cerebrum, Parietal Lobe, Inferior Parietal Lobule, Gray Matter, Brodmann area 40 |
| 32 | 0.006 | 0.221 | 45.57 | 5.16 | 0 | -20 -54 68 | Left Cerebrum, Parietal Lobe, Postcentral Gyrus,  Gray Matter, Brodmann area 7 |
| 12 | 0.024 | 0.644 | 38.30 | 4.85 | 0 | 50 -20 50 | Right Cerebrum, Parietal Lobe, Postcentral Gyrus, Gray Matter, Brodmann area 1 |
| 2 | 0.037 | 0.902 | 36.20 | 4.75 | 0 | 38 -32 58 | Right Cerebrum, Parietal Lobe, Postcentral Gyrus,  Gray Matter, Brodmann area 3 |
| 1 | 0.04 | 0.916 | 35.75 | 4.72 | 0 | 38 -22 54 | Right Cerebrum, Frontal Lobe, Precentral Gyrus,  Gray Matter, Brodmann area 4 |
| 1 | 0.046 | 0.925 | 35.11 | 4.69 | 0 | 34 -24 58 | Right Cerebrum, Frontal Lobe, Precentral Gyrus,  Gray Matter, Brodmann area 4 |
| 1 | 0.046 | 0.925 | 35.05 | 4.69 | 0 | -56 -48 44 | Left Cerebrum, Parietal Lobe, Inferior Parietal Lobule, Gray Matter, Brodmann area 40 |
|  |  |  |  |  |  |  |  |
| **Patients Visual Main Effects** | | |  |  |  |  |  |
|  |  |  |  |  |  |  |  |
| **cluster** | **peak** | **peak** | **peak** | **peak** | **peak** |  |  |
| **equivk** | **p(FWE-corr)** | **p(FDR-corr)** | **F** | **equivZ** | **p(unc)** | **x,y,z {mm}** | **Region** |
|  |  |  |  |  |  |  |  |
| 210 | 0.001 | 0.057 | 58.63 | 5.61 | 0 | 18 -56 0 | Right Cerebrum, Occipital Lobe, Lingual Gyrus,  Gray Matter, Brodmann area 19 |
|  | 0.004 | 0.15 | 47.81 | 5.24 | 0 | 16 -68 -6 | Right Cerebrum, Frontal Lobe, Superior Frontal Gyrus, Gray Matter, Brodmann area 10 |
|  | 0.022 | 0.44 | 38.78 | 4.87 | 0 | 24 -52 -12 | Right Cerebellum, Posterior Lobe, Declive |
| 58 | 0.006 | 0.15 | 46.12 | 5.18 | 0 | -18 -56 -6 | Left Cerebrum, Limbic Lobe, Parahippocampal Gyrus, Gray Matter, Brodmann area 19 |
|  |  |  |  |  |  |  |  |
|  |  |  |  |  |  |  |  |
| **Controls Vestibular Main Effects** | | |  |  |  |  |  |
|  |  |  |  |  |  |  |  |
| **cluster** | **peak** | **peak** | **peak** | **peak** | **peak** |  |  |
| **equivk** | **p(FWE-corr)** | **p(FDR-corr)** | **F** | **equivZ** | **p(unc)** | **x,y,z {mm}** | **Region** |
|  |  |  |  |  |  |  |  |
| 278 | 0 | 0.003 | 79.38 | 6.16 | 0 | 30 -98 -2 | Right Cerebrum, Occipital Lobe,  Middle Occipital Gyrus |
| 304 | 0 | 0.011 | 64.03 | 5.77 | 0 | -26 -98 -10 | Left Cerebrum, Occipital Lobe, Inferior Occipital Gyrus, Gray Matter, Brodmann area 17 |
|  | 0.002 | 0.056 | 51.01 | 5.36 | 0 | -26 -102 0 | Left Cerebrum, Occipital Lobe, Occipital Pole |
| 4 | 0.033 | 0.654 | 36.05 | 4.74 | 0 | -30 -50 -44 | Left Cerebellum, Posterior Lobe,  Cerebellar Tonsil |
|  |  |  |  |  |  |  |  |
| **Patients Vestibular Main Effects** | | |  |  |  |  |  |
|  |  |  |  |  |  |  |  |
| **cluster** | **peak** | **peak** | **peak** | **peak** | **peak** |  |  |
| **equivk** | **p(FWE-corr)** | **p(FDR-corr)** | **F** | **equivZ** | **p(unc)** | **x,y,z {mm}** | **Region** |
|  |  |  |  |  |  |  |  |
| 68 | 0 | 0.068 | 63.13 | 5.75 | 0 | 34 -44 2 | Right Cerebrum, Temporal Lobe, Sub-Gyral, Gray Matter, Hippocampus |
|  | 0.027 | 0.583 | 37.10 | 4.79 | 0 | 34 -54 6 | Right Cerebrum, Occipital Lobe, Lingual Gyrus,  Gray Matter, Brodmann area 19 |
| 88 | 0.001 | 0.085 | 57.19 | 5.57 | 0 | 30 -100 0 | Right Cerebrum, Occipital Lobe,  Middle Occipital Gyrus |
| 78 | 0.002 | 0.097 | 52.05 | 5.40 | 0 | -40 -48 -50 | Left Cerebellum, Posterior Lobe, Gray Matter |
| 60 | 0.01 | 0.299 | 42.47 | 5.03 | 0 | -28 -98 -10 | Left Cerebrum, Occipital Lobe, Inferior Occipital Gyrus, Gray Matter, Brodmann area 17 |
|  | 0.03 | 0.585 | 36.61 | 4.77 | 0 | -18 -98 -10 | Left Cerebrum, Occipital Lobe, Lingual Gyrus,  Gray Matter, Brodmann area 17 |
| 41 | 0.01 | 0.299 | 42.44 | 5.03 | 0 | -38 4 -38 | Left Cerebrum, Temporal Lobe, Middle Temporal Gyrus, Gray Matter, Brodmann area 38 |
| 10 | 0.015 | 0.356 | 40.18 | 4.93 | 0 | -28 -48 -44 | Left Cerebellum, Posterior Lobe,  Cerebellar Tonsil, Gray Matter |

**Supplementary Figure 1**


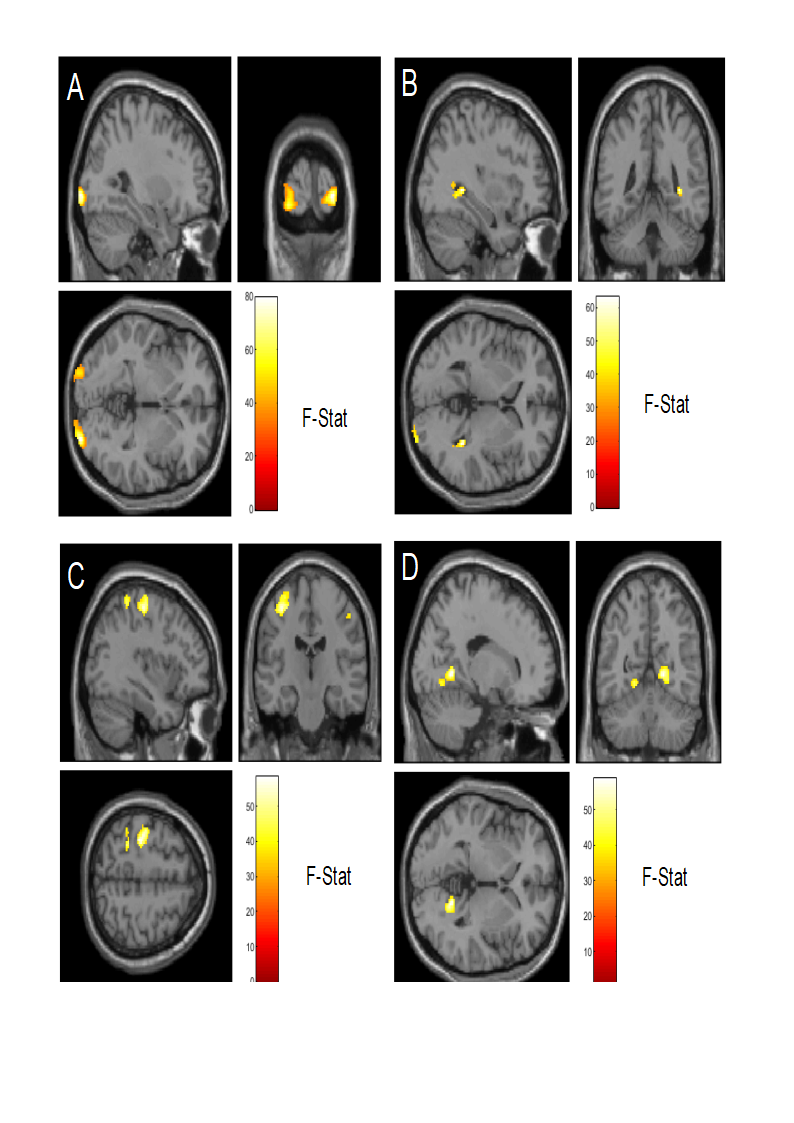


**Figure 1. Vestibular and Visual main effect activations for controls and patients.**

For main effects of vestibular stimulation: A) Controls displayed reductions in activation within primary visual cortex and occipital pole. B) Patients also displayed reductions in primary visual cortex, and increased activity within parahippocampal gyrus.

For main effects of visual stimulation: C) Controls displayed increases in primary motor, primary somatosensory and association cortices. D) Patients displayed reduced activity in the left anterior lobe culmen region; and the right lingual gyrus.

Activations are shown using F-statistics, whole brain FWE p<0.05 corrected for multiple comparisons.
